# Role of Adjuvants in Enhancing the Efficacy and Duration of Anesthesia Blocks: A Comprehensive Review

**DOI:** 10.7759/cureus.69880

**Published:** 2024-09-21

**Authors:** Akansha Singhal, Karuna Taksande

**Affiliations:** 1 Anesthesiology, Jawaharlal Nehru Medical College, Datta Meghe Institute of Higher Education and Research, Wardha, IND

**Keywords:** adjuvants, anesthesia blocks, local anesthetics, pain management, peripheral nerve blocks, regional anesthesia

## Abstract

Anesthesia blocks are integral to modern pain management, offering targeted and effective relief for various surgical and procedural interventions. These techniques, including regional and peripheral nerve blocks (PNBs), involve the administration of local anesthetics to specific body areas, either through epidural, spinal, or direct nerve injections. While effective, incorporating adjuvants, substances added to local anesthetics, can significantly enhance their efficacy and prolong their duration. Adjuvants such as opioids, corticosteroids, alpha-2 agonists, and nonsteroidal anti-inflammatory drugs (NSAIDs) are used to amplify analgesic effects, reduce the need for general anesthesia, and improve postoperative outcomes. This review explores the role of adjuvants in optimizing anesthesia blocks, examining their mechanisms of action, clinical benefits, and safety considerations. Adding adjuvants can lead to enhanced pain control, reduced dosage of local anesthetics, and fewer systemic side effects. By integrating adjuvants into anesthesia practice, clinicians can achieve more precise and sustained pain management, tailoring approaches to individual patient needs and specific procedural demands. This comprehensive review highlights current evidence on using adjuvants, their impact on anesthesia block effectiveness, and future research directions. Understanding the role of adjuvants is crucial for improving patient outcomes and advancing pain management techniques in various surgical settings.

## Introduction and background

Anesthesia blocks are a fundamental aspect of modern anesthesia practice, offering targeted pain relief for various surgical and procedural interventions [1]. These techniques involve the administration of local anesthetics to specific regions of the body to achieve effective analgesia, either by blocking sensory nerves or interrupting pain signal transmission to the central nervous system [2]. Regional blocks, for instance, include methods such as epidural and spinal anesthesia, which involve injecting anesthetics into areas around the spinal cord to cover larger areas of the body. On the other hand, peripheral nerve blocks (PNBs) target individual nerves or nerve bundles to provide pain relief to specific limbs or regions, such as the femoral nerve block used for knee surgeries or the sciatic nerve block for foot and ankle procedures [3]. The clinical significance of anesthesia blocks extends beyond mere pain management. They are commonly employed to reduce the necessity for general anesthesia, which can help minimize systemic side effects and shorten recovery times [4]. This targeted approach improves patient comfort during and after surgery and contributes to quicker postoperative recovery and reduced incidence of postoperative nausea. By effectively controlling pain and potentially enhancing patient outcomes, anesthesia blocks play a crucial role in various surgical disciplines, including orthopedics, obstetrics, and abdominal surgery [5].

Integrating adjuvants into anesthesia practice has significantly advanced the field by enhancing the efficacy and duration of anesthesia blocks. Adjuvants are substances added to local anesthetics to augment their effects, either by increasing the level of analgesia or by extending the duration of pain relief [6]. Their purpose is multifaceted: they can enhance the potency of local anesthetics, prolong the duration of analgesia, and improve overall pain management. By modulating pain pathways or working synergistically with local anesthetics, adjuvants help achieve more effective and sustained pain control [6]. In clinical practice, the role of adjuvants is pivotal in optimizing anesthesia techniques. Their use allows anesthesiologists to fine-tune pain relief strategies, potentially reducing the need for higher doses of local anesthetics and minimizing the risk of adverse effects [6]. By carefully selecting and administering adjuvants, healthcare providers can tailor anesthesia management to individual patient needs, improving the effectiveness of anesthesia blocks and the overall patient experience. This refined approach underscores the importance of understanding and leveraging adjuvants to enhance anesthesia outcomes and advance pain management practices [7].

## Review

Mechanism of action of anesthesia adjuvants

Anesthesia adjuvants play a pivotal role in enhancing the effectiveness of local anesthetics through various pharmacodynamic and pharmacokinetic interactions. These agents can impact the solubility and absorption of local anesthetics, resulting in a faster onset and extended duration of action [7]. For example, certain adjuvants can increase the solubility of local anesthetics, facilitating quicker absorption into target tissues. Additionally, vasoconstrictors like epinephrine are commonly used as adjuvants; they induce localized vasoconstriction, decreasing the local anesthetic's systemic absorption [7]. This not only prolongs the duration of anesthesia but also reduces the risk of systemic toxicity. Furthermore, many adjuvants exhibit synergistic analgesic effects when combined with local anesthetics, enhancing the quality and intensity of anesthesia [7]. Various types of adjuvants are used with local anesthetics, each offering distinct mechanisms of action and benefits. Opioids, such as morphine and fentanyl, are frequently employed to enhance analgesia. They act on mu-opioid receptors in the central nervous system, relieving pain. However, their use may be limited by side effects, including respiratory depression, nausea, vomiting, and pruritus, which require careful management [8]. Corticosteroids, particularly dexamethasone, have gained popularity for their ability to reduce inflammation and prolong analgesia. When administered perineurally, corticosteroids are more effective than systemic administration in extending the duration of pain relief, making them valuable in managing postoperative pain and other inflammatory conditions [9]. Alpha-2 agonists, such as clonidine and dexmedetomidine, are another class of adjuvants that enhance sedation and analgesia. These agents inhibit norepinephrine release, leading to a prolonged duration of sensory and motor blocks, significantly improving patient comfort during and after surgical procedures [10]. Nonsteroidal anti-inflammatory drugs (NSAIDs) are also employed as adjuvants because they reduce inflammation and pain by inhibiting the synthesis of prostaglandins. They are particularly effective in multimodal analgesia strategies, offering additional pain relief alongside local anesthetics [11]. Finally, other adjuvants, such as bicarbonate and vasoconstrictors, play important roles in anesthesia management. Adding bicarbonate to local anesthetics can increase the pH, enhance drug ionization, and lead to a faster onset of action. Vasoconstrictors, like epinephrine, extend the duration of anesthesia and help minimize systemic absorption, thereby reducing the risk of toxicity [12].

Clinical evidence on efficacy and duration

Effectiveness of Common Adjuvants

Numerous studies and meta-analyses have explored the role of various adjuvants in enhancing the effects of local anesthetics. Among the most extensively studied is dexamethasone, a corticosteroid renowned for its anti-inflammatory properties [7,13]. Research indicates that dexamethasone can significantly extend the duration of analgesia in PNBs, with some studies reporting an increase in analgesic effects by up to 50% compared to local anesthetics used alone. This makes dexamethasone a valuable addition to regional anesthesia protocols, especially for patients undergoing procedures associated with inflammatory pain [14,15]. Another prominent adjuvant is clonidine, an alpha-2 adrenergic agonist. Clonidine has been shown to enhance the duration of sensory and motor blocks, with meta-analyses indicating an average increase in analgesia duration of approximately 30-40%. Its dual action-providing sedation while prolonging analgesia makes it particularly appealing for use in regional anesthesia. Similarly, dexmedetomidine, a more selective alpha-2 agonist, has gained attention for its ability to extend analgesia while maintaining a favorable safety profile. Studies suggest that dexmedetomidine can enhance the quality of anesthesia and prolong sensory block duration without significant adverse effects [16-18]. Magnesium has also emerged as a noteworthy adjuvant in anesthesia. As an NMDA receptor antagonist, magnesium can potentially reduce central sensitization, a key factor in the development of chronic pain. While the efficacy of magnesium as an adjuvant is still being explored, some studies suggest that it can contribute to prolonged analgesia when used alongside local anesthetics [19]. When comparing the efficacy of these adjuvants, it becomes clear that while all enhance analgesic effects, their effectiveness varies significantly. Dexamethasone is often regarded as one of the most effective adjuvants for prolonging nerve block duration, particularly in inflammatory settings. In contrast, clonidine and dexmedetomidine are favored for their sedative properties, making them ideal for more complex regional anesthesia scenarios. However, magnesium shows promise but is less frequently utilized than other adjuvants due to the variability in outcomes reported across different studies [13].

Duration of Anesthesia

The impact of adjuvants on extending the duration of anesthesia blocks has been a major focus of clinical research. The incorporation of adjuvants has consistently proven to significantly prolong anesthesia block effectiveness. For instance, research indicates that dexamethasone can extend nerve block duration by several hours, substantially reducing the need for rescue analgesia during the postoperative period. This extension enhances patient comfort and contributes to a more favorable recovery experience [20]. Similarly, the use of clonidine and dexmedetomidine has been associated with extended sensory and motor block durations. Clinical trials have reported increases in the duration of action by 50% or more when these adjuvants are combined with local anesthetics. These findings highlight the importance of selecting appropriate adjuvants to optimize analgesic outcomes for patients undergoing surgical procedures [21]. Numerous case studies and clinical trials further support the efficacy of these adjuvants. For example, a clinical trial involving dexamethasone in brachial plexus blocks demonstrated a significant increase in analgesia duration, with patients experiencing lower pain scores and longer intervals before needing additional analgesics. Similarly, studies involving clonidine and dexmedetomidine have shown that patients benefit from prolonged pain relief and reduced opioid consumption postoperatively, underscoring the role of these adjuvants in multimodal analgesia strategies [22].

Safety and side effects

Using adjuvants in combination with local anesthetics can lead to various adverse effects. Common side effects include local reactions such as pain, swelling, or erythema at the injection site and systemic reactions like fever, malaise, or fatigue, which are generally mild and transient [7]. Rare but serious side effects may include neurotoxicity, potentially resulting in nerve injury; however, this is infrequent and usually associated with specific adjuvants or high concentrations. Allergic reactions, including anaphylaxis or other hypersensitivity reactions, can occur, though rare. Some adjuvants have also been linked to a risk of developing autoimmune disorders, though definitive causation remains unclear [7]. Certain patient populations may be at higher risk for adverse effects from adjuvants. Immunocompromised individuals, those with weakened immune systems, may experience different reactions. Patients with a history of allergies to medications or vaccines may be more susceptible to adverse effects. Older adults or those with multiple health conditions might face increased risks due to altered pharmacodynamics and pharmacokinetics [23]. Effective monitoring of patients receiving adjuvants requires a pre-administration assessment to evaluate the patient's history of allergies and previous reactions to adjuvants or local anesthetics. Post-administration monitoring is crucial, especially within the first hour, as this is when most acute side effects are likely to manifest. Detailed documentation of any adverse effects experienced by patients is essential for future reference and to inform ongoing safety evaluations [7]. Optimizing the dose is crucial to minimize risks associated with adjuvants. Using the lowest effective dose to achieve the desired effect can help reduce the likelihood of adverse reactions. Patient education is also important; informing patients about potential side effects and reporting any unusual symptoms post-administration is essential. For patients with known sensitivities or those at high risk for adverse effects, considering alternative adjuvants or techniques that pose less risk is recommended [24]. The safety and side effects of common adjuvants used in anesthesia blocks are summarized in Table 1.

**Table 1:** Safety and side effects of common adjuvants used in anesthesia blocks

Adjuvant	Common Side Effects	Severe Adverse Reactions	Reported Incidence	Notes on Safety
Epinephrine [25]	Increased heart rate, tremors	Myocardial ischemia, arrhythmias	Low	Generally safe in low doses, monitor cardiac patients carefully.
Dexmedetomidine [26]	Bradycardia, hypotension	Severe hypotension, rebound hypertension	Moderate	Use with caution in patients with cardiovascular conditions.
Clonidine [27]	Hypotension, sedation	Severe hypotension, bradycardia	Moderate	It can prolong the block duration significantly but requires careful monitoring.
Ketamine [28]	Dizziness, nausea	Hallucinations, increased intracranial pressure	Low	Effective for pain relief but can cause psychomimetic effects in some patients.
Magnesium Sulfate [29]	Flushing, hypotension	Respiratory depression, muscle weakness	Low	Considered safe in controlled doses, careful monitoring of respiratory function is required.
Buprenorphine [30]	Nausea, vomiting, dizziness	Respiratory depression, severe bradycardia	Low	Long-lasting effects, but respiratory depression is a concern in sensitive patients.
Dexamethasone [31]	Hyperglycemia, mood changes	Immunosuppression, peptic ulcers	Moderate	Well-tolerated but should be avoided in diabetic and immunocompromised patients.
Midazolam [32]	Sedation, respiratory depression	Apnea, paradoxical reactions	Moderate	Short-acting but can cause significant respiratory issues in higher doses.

Practical considerations in clinical practice

When integrating adjuvants into anesthesia practice, careful consideration of dosage, administration techniques, and patient-specific factors is essential to achieve optimal outcomes and minimize risks associated with anesthesia blocks [7]. The recommended dosages of local anesthetics depend on the specific agent used and the type of block performed. For example, bupivacaine is commonly administered as a 0.25% to 0.5% solution, with a maximum dosage of 175 mg for PNBs. Ropivacaine, another frequently used local anesthetic, is given in concentrations ranging from 0.2% to 0.75%, with a maximum of 200 mg for PNBs [33]. Adjuvants also have specific recommended dosages: dexamethasone is typically used at doses of 4-10 mg perineurally, clonidine at 1-2 mcg/kg (not exceeding 150 mcg per block), and dexmedetomidine and magnesium at dosages of 0.5-1 mcg/kg and 30-50 mg/kg, respectively [7]. Administration techniques are critical for the effectiveness of adjuvants. Perineural administration, which involves direct injection around the nerve, can enhance local effects and minimize systemic absorption. When combining adjuvants with local anesthetics, ensuring compatibility to prevent precipitation or degradation of the solutions is essential [7]. Maintaining the total injection volume within safe limits is important to minimize tissue trauma and ensure effective spread. Continuous monitoring of patients for signs of adverse reactions, particularly with opioids or alpha-2 agonists, is crucial for patient safety. Proper documentation of dosages and combinations used, along with the rationale for their selection, ensures continuity of care and aids in future treatment decisions [34]. Patient-specific factors significantly influence the use of adjuvants in anesthesia. Age is key; pediatric patients often require dosage adjustments due to metabolism and body composition differences, necessitating smaller volumes and lower concentrations. Conversely, elderly patients may show increased sensitivity to anesthetics and adjuvants, requiring careful dosage adjustments and close monitoring for prolonged effects [35]. Comorbidities also play a significant role; patients with cardiovascular conditions may be more susceptible to the effects of certain adjuvants, such as clonidine and dexmedetomidine, which can cause bradycardia or hypotension. Renal or hepatic impairment can affect drug clearance, further necessitating dosage adjustments and vigilant monitoring [36]. Obesity is another important consideration, as dosage adjustments may be required based on lean body mass, especially for lipophilic agents like bupivacaine. A thorough preoperative assessment is essential to identify these patient-specific factors that may influence the choice and dosage of adjuvants [37]. Tailoring the use of adjuvants based on a patient’s pain history, previous responses to anesthesia, and specific surgical procedures is crucial for optimizing outcomes. A multimodal analgesia approach can enhance pain management, including non-pharmacological methods such as physical therapy and cognitive-behavioral strategies [38]. Practical considerations for the use of adjuvants in clinical anesthesia practice are summarized in Table 2.

**Table 2:** Practical considerations for the use of adjuvants in clinical anesthesia practice

Adjuvant	Optimal Dosage Range	Onset of Action	Duration of Action	Clinical Application	Special Considerations
Epinephrine [2]	5-20 mcg/mL	Rapid (within minutes)	Short (10-20 minutes)	Prolongs local anesthetic effect reduces bleeding	Use cautiously in cardiac patients; avoid in-end arteries.
Dexmedetomidine [39]	0.5-1 mcg/kg	10-15 minutes	Long (up to 6 hours)	Enhances analgesia, reduces opioid requirements	It may cause hypotension and bradycardia, especially in the elderly.
Clonidine [40]	0.5-2 mcg/kg	15-30 minutes	Long (6-8 hours)	Prolongs anesthesia duration, provides postoperative pain relief	Monitor for hypotension and bradycardia, especially in prolonged use.
Ketamine [41]	0.1-0.5 mg/kg	Rapid (within minutes)	Short (30-60 minutes)	Effective for short procedures, reduces opioid consumption	It may cause psychomimetic effects; avoid it in patients with elevated ICP.
Magnesium Sulfate [42]	30-50 mg/kg	20-30 minutes	Moderate (up to 2 hours)	Potentiates local anesthetics, reduces pain	Monitor serum magnesium levels to prevent toxicity.
Buprenorphine [30]	0.1-0.3 mg	15-30 minutes	Very long (up to 12 hours)	Long-lasting postoperative analgesia	Risk of respiratory depression, avoid in opioid-tolerant patients.
Dexamethasone [13]	4-10 mg	10-20 minutes	Long (12-24 hours)	Reduces inflammation and prolongs block duration	Avoid in diabetic patients due to the risk of hyperglycemia.
Midazolam [32]	1-2 mg	5-10 minutes	Short (2-4 hours)	Adjunct for sedation and anxiety relief	Risk of respiratory depression in high doses or prolonged use.

Future directions and research

The field of adjuvant research is rapidly evolving, with several new adjuvants and innovative delivery systems being explored to enhance the efficacy and duration of anesthesia blocks. Notable trends include the development of next-generation adjuvants that target pathways beyond traditional pattern recognition receptors (PRRs). These new adjuvants leverage mechanisms such as metabolic alterations, epigenetic modifications, and the release of damage-associated molecular patterns (DAMPs) to elicit a more robust immune response [43]. Advances in materials science are contributing to the creation of adjuvants that incorporate molecular adjuvants, polymers, lipids, and inorganic nanoparticles. These developments aim to improve antigen delivery and enhance immune responses, particularly in challenging fields such as cancer immunotherapy. Additionally, the use of multiple adjuvants in a single formulation is gaining momentum due to its potential to create synergistic effects that boost both humoral and cellular immunity, which is particularly beneficial for vaccines targeting complex pathogens. The focus is also shifting toward synthetic adjuvants that can be produced at scale with consistent quality, offering advantages over traditional natural components in terms of reduced variability and improved safety profiles [44]. Despite these advancements, several gaps remain in the understanding and applying adjuvants in anesthesia. There is a pressing need for a more systematic exploration of how different adjuvants enhance local anesthetics. A deeper understanding of these mechanisms could lead to the rational design of adjuvant combinations tailored to specific clinical scenarios. Many promising adjuvants are still preclinical, and their safety and efficacy in human subjects require thorough investigation. Future research should prioritize clinical trials to validate the effectiveness of new adjuvants and delivery systems in real-world settings [7]. Given the variability in patient responses to anesthesia, personalized medicine approaches could be advantageous. Investigating how individual genetic and immunological profiles affect adjuvant responses could lead to more effective and tailored anesthetic strategies. Moreover, the development of adjuvants should consider sustainability in sourcing materials and the feasibility of large-scale production, as addressing these factors will be crucial for ensuring that new adjuvants are accessible and practical for widespread clinical use [45]. Future directions and research in the use of adjuvants for anesthesia blocks are summarized in Table 3.

**Table 3:** Future directions and research in the use of adjuvants for anesthesia blocks

Area of Research	Key Focus	Potential Benefits	Challenges/Limitations	Current Status
Novel Adjuvants [46]	Development of new adjuvants with minimal side effects	Enhanced safety and efficacy, longer block duration	Limited clinical trials, regulatory hurdles	Early-phase trials and preclinical studies
Personalized Medicine in Anesthesia [45]	Tailoring adjuvant use based on patient genetics	Optimized dosing, reduced side effects, individualized care	Need for genetic testing infrastructure, cost concerns	Ongoing research in pharmacogenomics
Long-Term Safety Studies [47]	Assessing long-term effects of adjuvant use	Better understanding of chronic side effects	Requires long-term follow-up and large patient cohorts	Few long-term studies are available
Combination Therapies [48]	Exploring the synergistic effects of multiple adjuvants	Increased efficacy, reduced doses of individual adjuvants	Complex interactions, risk of enhanced side effects	Preliminary studies showing promise
Adjuvants for Regional Anesthesia in Special Populations [49]	Focusing on geriatric, pediatric, and high-risk patients	Improved outcomes in vulnerable populations	Higher risk of adverse events need for cautious dosing	Research in specialized patient groups is growing
Mechanisms of Action [50]	Understanding how adjuvants modulate nerve conduction	Insights into improving effectiveness and reducing side effects	Mechanistic studies can be complex and require advanced technology	Research progressing but incomplete
Non-Pharmacological Enhancements [51]	Integration of adjuvants with non-pharmacological techniques (e.g., ultrasound)	Enhanced precision, reduced drug dosage	Requires specialized training and equipment	Growing interest, especially in advanced clinical settings
AI and Machine Learning in Adjuvant Selection [52]	Using AI to predict optimal adjuvants and dosing	Personalized regimens, reduced adverse reactions	Requires large data sets, algorithm transparency	Emerging field with ongoing pilot studies

## Conclusions

In conclusion, integrating adjuvants into anesthesia practice represents a significant advancement in managing pain during and after surgical procedures. By enhancing the efficacy and extending the duration of anesthesia blocks, adjuvants improve the quality of pain relief and contribute to a more tailored and patient-centered approach to anesthesia. Their ability to reduce the need for higher doses of local anesthetics and mitigate systemic side effects underscores their value in optimizing anesthesia outcomes. As research continues to evolve and new adjuvants are explored, the potential for further enhancing pain management and improving patient experiences remains promising. Ultimately, the judicious use of adjuvants will continue to play a crucial role in refining anesthesia techniques, ensuring safer and more effective pain control for diverse clinical scenarios.
